# Recent advances in the development of nature-derived photocrosslinkable biomaterials for 3D printing in tissue engineering

**DOI:** 10.1186/s40824-019-0168-8

**Published:** 2019-11-19

**Authors:** Geunho Choi, Hyung Joon Cha

**Affiliations:** Department of Chemical Engineering, Pohang University of Science and Technology, Pohang, 37673 South Korea

**Keywords:** 3D printing, Photocrosslinking, Biomaterials, Tissue engineering

## Abstract

**Background:**

In recent years, three-dimensional (3D) printing has begun to be widely used in tissue engineering. Natural biomaterials have been employed to overcome the limitations of synthetic polymers. However, their low mechanical strength and poor printability are major disadvantages. Photocrosslinking is the most promising fabrication strategy because it is non-invasive and easy to control light intensity and exposure. In this article, developments of photocrosslinkable natural biomaterials in the field of 3D printing are reviewed.

**Main body:**

Photocrosslinkable biomaterials can be broadly classified into materials that use ultraviolet (UV) and visible lights. Many natural biomaterials such as gelatin, hydroxyapatite, silk fibroin, and pectin have been modified through acrylation, crosslinked by 365 nm UV light, and 3D printed. Riboflavin could also be used to crosslink and print collagen or decellularized extracellular matrix (dECM). In the case of silk-like aneroin and modified gelatin, crosslinking is possible by forming a dityrosine bond using 452 nm visible light.

**Conclusion:**

Despite the tremendous researches on the developments of photocrosslinkable 3D printing natural biomaterials, further efforts are necessary to develop source biomaterials with excellent biological functions and sufficient mechanical integrity.

## Introduction

Three-dimensional (3D) printing in tissue engineering field is a fast and solid construction method for highly automated and reproducible production of 3D structural bioscaffolds. This is a technique that can solve the spatio-temporal placement of biomaterials, cells, and many functional materials, which was difficult with conventional tissue engineering methods [1]. 3D printing requires 3D design through a computer and construction of structures through various printing methods. The most commonly used 3D printing methods are extrusion, ink-jet, and light-assisted printings [2, 3]. For extrusion 3D printing, the resolution is much lower than other methods. Ink-jet 3D printing is generally limited to low viscosity materials [4]. Light-assisted 3D printing is costly and the available material constraints are very large [5, 6]. Therefore, researchers need to select a suitable printing method depending on physical properties of 3D printing materials.

**Fig. 1:**
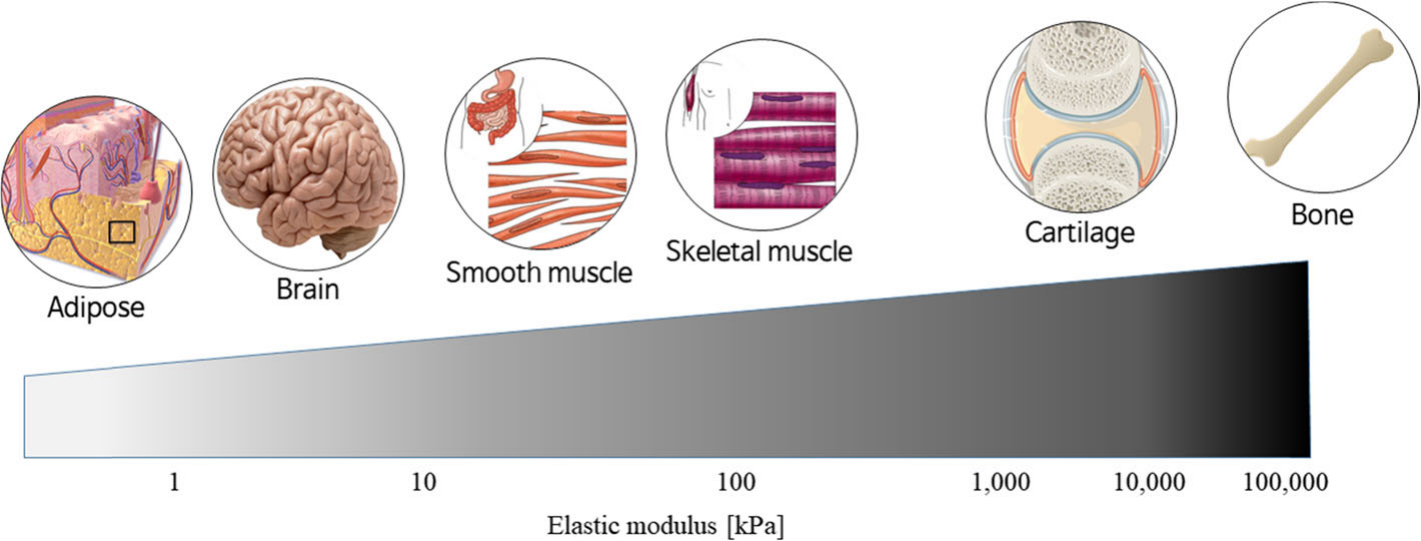
Elastic modulus of native tissues and organs [13, 14]

To be used as an ideal 3D printing material, it should have sufficient mechanical property and structural integrity, but at the same time, it needs excellent biological characteristics [7]. While many synthetic polymers have been widely used as 3D printing materials, they only provide sufficient mechanical properties but cannot have biological functions [8, 9]. Thus, they can only be used in limited tissue engineering areas such as implants. To overcome the limitations of synthetic polymers, natural biomaterials have begun to be considered. However, despite their excellent biocompatibility, it is difficult to make 3D structures due to their insufficient printability and mechanical integrity [10, 11, 12]. In addition, cell behaviors are greatly affected by mechanical properties of 3D structural bioscaffolds. Thus, proper cell types should be used depending on the mechanical properties of 3D structures to mimic the actual native tissues and organs (Fig. 1) [13, 14]. Lots of researchers have been struggling to develop suitable 3D printing materials with high printability and excellent biocompatibility.

**Table 1:** Several parameters of recently developed nature-derived photocrosslinkable biomaterials for 3D printing

Major biomaterial(s)		Photoinitiator	Curing parameter		Shape fidelity		Printing resolution	Printing method /Construct size	Cell type	Reference
			Intensity [mW /cm^2^]	Curing time [sec]	Compressive modulus	Storage modulus				
UV light-based	HAMA & GelMA	Irgacure 2959	N/A	30, 90	~ 9 kPa	N/A	800 μm	Extrusion /10 mm × 10 mm × 1.2 mm	BAP, WAP	33
	PECMA	Irgacure 2959	7	160	N/A	~ 2.6 kPa	N/A	Extrusion /8 mm × 8 mm × 4.5 mm	hNDFs	35
	SilMA	LAP	30	4	~ 125 kPa	~ 25 kPa	66 μm (circle) 90 μm (square) 142 μm (height)	Light-assisted /25 mm × 25 mm × 50 mm	Chondrocyte	5
	Collagen	Riboflavin	1200	10	N/A	~ 2 kPa	900–1100 μm	Extrusion /25 mm × 25 mm × 0.4 mm	Chondrocyte	39
	dECM	Riboflavin	30	180	~ 15 kPa	10–30 kPa	114–860 μm	Extrusion /N/A	CPCs	41, 44
Visible light-based	Aneroin	Ru(II)bpy_3_^2+^	2000	10	~ 6400 kPa	N/A	100–950 μm	Extrusion /35 mm × 50 mm × 18 mm	MSCs	12
	Gtn-HPA	Ru(II)bpy_3_^2+^	N/A	10, 20, 30	~ 55 kPa	N/A	N/A	Extrusion /N/A	COS-7	49

Abbreviation: *HAMA* methacrylated hyaluronic acid, *GelMA* methacrylated gelatin, *PECMA* Pectin methacrylate, *SilMA* chemically modified silk fibroin by glycidyl methacrylate; *dECM* decellularized extracellular matrix, Aneroin, sea anemone-derived silk-like protein, *Gtn-HPA* gelatin-hydroxyphenylpropionic acid conjugate *LAP* lithium phenyl(2,4,6-trimethylbenzoyl) phosphinate, *N/A* not available, *BAP* brown adipose progenitor cells, *WAP* white adipose progenitor cells, *hNDFs* human neonatal dermal fibroblasts, *CPCs* cardiac progenitor cells, *MSCs* mesenchymal stem cells; *COS-7* kidney cells derived from the African green monkey

One approach to solve the problem of insufficient printability and mechanical integrity of natural biomaterials is the use of the additional crosslinking strategy. Among many crosslinking strategies, photocrosslinking has been considered as the most promising method to allow rapid and durable curing by forming additional intra−/inter-molecular chemical bonds [15]. In this article, we reviewed the types of photocrosslinkable 3D printing natural biomaterials and the recent advances in their developments (summarized in Table 1).

## Major 3D printing strategies in tissue engineering

### Extrusion 3D printing

Extrusion method is the most widely used 3D printing technology in recent decade. This method pushes a printing material using pneumatic, piston, or screw-drive. The biggest advantage of extrusion is that any materials can be used if they have sufficient viscosity [16]. Moreover, 3D printing can be easily implemented without a deep understanding of the technology [17]. However, despite this convenience and versatility, there are also some disadvantages compared to other technologies. The biggest drawback is low resolution, which typically cannot be deposited more precisely than 100 μm, resulting in a rough surface [18].

### Ink-jet 3D printing

Ink-jet printing dispenses droplets from thermal or piezoelectric actuator and generates 3D structures. The advantages of ink-jet are relatively low cost, fast printing process, and high resolution [16]. However, limited range of printing materials and their low mechanical properties are major disadvantages [19]. Because surface tension cannot be overcome and droplets cannot be formed when viscosity is too high, only ink with a viscosity of less than 30 cP can be available [20, 21]. Due to the low viscosity conditions, ink concentration is also low, resulting in difficult formation of stacks or solid layers.

### Light-assisted 3D printing

Light-assisted 3D printing systems can be categorized into two subgroups: digital light processing (DLP)- and laser-based printings [22]. In the case of DLP method, it is very similar to stereolithography. A light, such as ultraviolet (UV), is exposed to a photopolymerizable pre-polymer solution and produces a 3D scaffold in layer-by-layer form [23]. For laser-based printing, donor-slide contains a printing material and laser radiation absorbing layer. When laser pulse is focused on donor-slide, droplets are formed by evaporation and deposited on collector-slide [24, 25]. Common advantages of light-assisted printing are high resolution and fast processing, and does not require supporting material. However, the disadvantages are high cost and large constraints of available printing materials (only photopolymerizable materials can be used) [5, 6, 18].

## Key advantages and disadvantages of photocrosslinking

Photocrosslinking has several advantages over other crosslinking methods. The accuracy of printed structures can be of great benefit. If a 3D printing material does not solidify quickly after exiting nozzle, it will be dispersed and finally results in lower resolution. Also, while high temperature or different pH may liquate the existing layer, generally light does not. Light is easily adjustable for application and intensity. In addition, partial exposure by position control is also possible. Controlling the printing process is easy because it is not restricted by mixing time and gelation time and does not depend on other variables. It is also possible to adjust curing speed and curing degree of output structure as required [26].

Nevertheless, there are some disadvantages at the same time. First, the types of photopolymerizable functional groups that originally exist in natural biomaterials are very limited. Although photopolymerizable functional groups can be introduced through chemical conjugations, there is a high possibility that cytotoxicity might be induced by newly attached chemical groups. In addition, the number of water-soluble and non-cytotoxic photoinitiators is very limited [27].

### UV light-based photocrosslinkable biomaterials

UV is the most widely used light source for photocrosslinkable materials. The most widely used method for imparting UV curing property is acrylation. Most of the biomaterials, including methacrylated gelatin (GelMA), have been conjugated with acrylate for photocrosslinking. Typically, methyl methacrylate (MA) or glycidyl methacrylate (GMA) is used to make C=C double bonds in target biomaterials [28]. Photocrosslinking can be initiated by dissolving the photoinitiator in the prepared material and exposing UV at a wavelength of 365 nm. Unfortunately, the disadvantage of acrylation is that biocompatibility can be impaired. After photocrosslinking, unreacted acryl groups can cause an inflammatory reaction [4, 29]. In addition, there is the possibility of cytotoxicity and potential gene mutation effects by UV radiation [30]. Irgacure 2959, the most commonly used photoinitiator, is cytotoxic [31]. Thus, researches on the development of more cell-friendly photoinitiators are underway, but there are still disadvantages such as low efficiency and high synthesis cost.

### Acrylated biomaterials

Gelatin is a protein that is obtained by hydrolyzing and purifying natural proteins composed of animal skins, cartilage, tendons, and the like. Gelatin is one of the substances present in extracellular matrix (ECM), which can improve cell adhesion and support cell growth and biological function due to its properties like RGD motif [32]. However, for 3D printing, pure gelatin is difficult to be used because of its mechanical properties. Gelatin is very sensitive to temperature; generally exists as a gel at a temperature below 35 °C but forms a liquid at a higher temperature. Therefore, when the physiological temperature is maintained, it exists as a liquid with low viscosity [33]. To overcome this problem, GelMA was developed in the 2000s. GelMA has attracted much attention in the field of tissue engineering due to its good bioactivity and physico-chemical properties which were achieved by UV light-mediated photocrosslinking [34]. Numerous studies have already been conducted using GelMA and its commercialization was achieved.

After the successful development of GelMA, various methacrylated biomaterials, such as methacrylated hyaluronic acid (HAMA), chemically modified silk fibroin by glycidyl methacrylate (SilMA), and pectin methacrylate (PECMA), have been developed. Hyaluronic acid (HA) is an anionic glycosaminoglycan that is abundant in ECM. It has a very high viscosity and a molecular weight of several million daltons in vivo according to sources [35]. HAMA based on HA was synthesized to allow the formation of photocrosslinkable hydrogel. It was successful in printing HAMA/GelMA hydrogels containing brown adipose tissue and white adipose progenitor cells [36, 37].

PECMA based on pectin was developed to allow double crosslinkings by UV light and CaCl_2_ [38]. Biofunctionalization of PECMA was also reported by fusion with cell adhesive peptide RGD. RGD-PECMA was able to be printed with human neonatal dermal fibroblasts, and it was confirmed that it maintained cell function even after 14 days of incubation.

Silk fibroin-based SilMA was also developed with the use of photoinitiator, lithium phenyl(2,4,6-trimethylbenzoyl) phosphinate (LAP), which has higher water solubility and lower cytotoxicity than irgacure 2959 [5]. SilMA was proper for laser-assisted printing because its viscosity is relatively low but the mechanical properties of the final printed structure are high. It showed a good resolution that can be up to 66 μm depending on the shape and the integrity of the printing structure was also very good. Its short-term and long-term biocompatibility experiments were also verified. The cytotoxicity of NIH/3 T3 cells was confirmed for 14 days and there was no significant difference compared to the case of GelMA. In addition, SilMA-based ring-shaped cartilage-like tissue containing human chondrocytes was observed for 4 weeks and provided a good environment for chondrocyte survival and cartilage formation.

### Non-acrylated biomaterials

Another photoinitiator for UV curing is riboflavin, also called vitamin B2. The main advantage of riboflavin is that it naturally exists in the body, unlike other photoinitiators, and is not cytotoxic [39]. Riboflavin can cause covalent binding of collagen and proteoglycan core proteins through UV irradiation [40]. Riboflavin-mediated photocrosslinkable collagen was developed [41, 42] and its printability and shape fidelity were investigated [43]. However, because its reaction time was too long and mechanical properties and resolution were still very low, it was difficult to make multilayered 3D structures [41].

One of the natural biomaterials that have recently been attracting much attention is decellularized ECM (dECM). This biomaterial is capable of dynamically interacting with cells and can affect cell migration, proliferation, and differentiation. dECM has the advantage that it can provide almost the same microenvironment as natural tissues in 3D microstructure [35, 44]. However, if complete decellularization cannot be achieved, these advantages might be completely negated in vivo. In addition, possible contaminated cellular DNAs might cause an immune response. Thus, the establishment of optimal decellularization process is important; too strong process can also cause severe damage to ECM materials [45, 46]. 3D printing using riboflavin-mediated dECM was attempted with mixing cardiac precursor cells, and the print resolution was adjusted from 114 to 860 μm by controlling parameters [47]. Each layer was printed with a curing time of 3 min and succeeded in printing up to 10 layers. After culturing the printed 3D constructs for 7 days, high survival and proliferation of cardiac precursor cells were observed and differentiation into myocardial cells was also confirmed.

### Visible light-based photocrosslinkable biomaterials

Another promising type of photocrosslinking is the use of phenolic residues in target natural biomaterials. Tyrosine, one of the 20 standard amino acids, has a phenol functionality. There have been attempts to form hydrogels through the formation of dityrosine bonds, a crosslinked form between two tyrosines, using horseradish peroxidase and H_2_O_2_. Dityrosine bonds also can be obtained even when iron ions are used to cause Fenton reaction [48].

Recently, there has been an attempt to artificially produce these dityrosine bonds using photoinitiation. Tris(2,2′- bipyridine)ruthenium(II) (Ru(II)bpy_3_^2+^; Rubpy), a photoinitiator, and persulfate, an electron acceptor, can generate radicals using blue light of 405 nm wavelength [49]. The advantage of this reaction is that it can avoid the potential mutation and phototoxicity because it uses safe visible light [15]. The very fast reaction rate is also another advantage. In addition, there is no need for chemical conjugation of functional groups on target biomaterials. However, because the radical reaction is explosive, there is a high risk of instantaneous cytotoxicity during the reaction [50]. Although most persulfates disappear in the process of forming dityrosine bonds, remained persulfates may be toxic if not completely consumed during the reaction [51]. Therefore, optimizations of crosslinking time and crosslinking agent concentration are necessary.

Materials that can use visible light include gelatin-hydroxyphenylpropionic acid conjugate (Gtn-HPA) and aneroin. Gtn-HPA is a biomaterial conjugated with a substance having a phenolic functional group and was developed as a 3D printing material that can be cured with visible light using Rubpy [52]. This material was able to allow survival of more than 85% COS-7 cells after 3 days.

Aneroin is a silk-like repetitive protein derived from sea anemone [53]. It has superior biodegradability compared to silk fibroin while showing excellent mechanical properties by β-spiral secondary structures. Aneroin is genetically producible and, unlike naturally extracted biomaterials directed from organisms, it can have uniform properties in molecular weight and the like. In addition, high tyrosine content is advantageous for dityrosine photocrosslinking. Aneroin was developed as a 3D printing material by mixing with HA to improve viscosity and introduce biological function [15]. The resolution was freely adjustable between 100 μm and 950 μm. Without sacrificial layers, complex 3D structures were successfully constructed. It showed excellent compression modulus of up to 6.42 MPa at 40% strain. It was also demonstrated good cell compatibility for 4 cell lines including mesenchymal stem cells. Unlike silk fibroin-based constructs, multinuclear giant cells were not found in the aneroin-based 3D constructs, indicating that the immune response was not induced.

## Conclusion

One of the important goals for 3D printing research in tissue engineering area is to develop ideal 3D printing materials that can have both excellent biocompatibility and high printability. Additional crosslinking approach has been utilized to overcome low mechanical property problem when using natural biomaterials. Photocrosslinking strategy is easy to control and non-invasive. In addition, it can be performed at mild conditions of temperature and pH. Thus, various types of nature-derived photocrosslinkable biomaterials have been developed. Despite the tremendous efforts on the development of photocrosslinkable 3D printing biomaterials, there are still many challenges to increase printability, biocompatibility, biofunctionality, biodegradability, and scalability.

## Data Availability

Please contact author for data requests.

## References

[1] Park J, Gao G, Jang J, Cho D (2016). 3D printed structures for delivery of biomolecules and cells: tissue repair and regeneration. J Mater Chem B.

[2] Dorishetty P, Balu R, Sreekumar A (2019). Robust and tunable hybrid hydrogels from photo-cross-linked soy protein isolate and regenerated silk fibroin. ACS Sustain Chem Eng.

[3] Mandrycky C, Wang Z, Kim K, Kim D (2016). 3D bioprinting for engineering complex tissues. Biotechnol Adv.

[4] Parak A, Pradeep P, du Toit L, Kumar P, Choonara Y, Pillay V (2019). Functionalizing bioinks for 3D bioprinting applications. Drug Discov Today.

[5] Kim S, Yeon Y, Lee J, et al. Precisely printable and biocompatible silk fibroin bioink for digital light processing 3D printing. Nat Commun. 2018;9(1). 10.1038/s41467-018-03759-y. 10.1038/s41467-018-03759-y PMC5915392 29693652

[6] Hollister S (2005). Porous scaffold design for tissue engineering. Nat Mater.

[7] DeSimone E, Schacht K, Jungst T, Groll J, Scheibel T (2015). Biofabrication of 3D constructs: fabrication technologies and spider silk proteins as bioinks. Pure Appl Chem.

[8] Yao Q, Wei B, Liu N (2015). Chondrogenic regeneration using bone marrow clots and a porous Polycaprolactone-hydroxyapatite scaffold by three-dimensional printing. Tissue Eng A.

[9] Li Z, Torgersen J, Ajami A (2013). Initiation efficiency and cytotoxicity of novel water-soluble two-photon photoinitiators for direct 3D microfabrication of hydrogels. RSC Adv.

[10] Jeon O, Lee Y, Hinton T, Feinberg A, Alsberg E (2019). Cryopreserved cell-laden alginate microgel bioink for 3D bioprinting of living tissues. Mater Today Chem.

[11] Pereira R, Bártolo P (2015). 3D bioprinting of photocrosslinkable hydrogel constructs. J Appl Polym Sci.

[12] Panwar Amit, Tan Lay (2016). Current Status of Bioinks for Micro-Extrusion-Based 3D Bioprinting. Molecules.

[13] Butcher Darci T., Alliston Tamara, Weaver Valerie M. (2009). A tense situation: forcing tumour progression. Nature Reviews Cancer.

[14] Handorf Andrew M, Zhou Yaxian, Halanski Matthew A, Li Wan-Ju (2015). Tissue Stiffness Dictates Development, Homeostasis, and Disease Progression. Organogenesis.

[15] Park T, Yang Y, Ha D, Cho D, Cha H (2019). Marine-derived natural polymer-based bioprinting ink for biocompatible, durable, and controllable 3D constructs. Biofabrication.

[16] Derakhshanfar S, Mbeleck R, Xu K, Zhang X, Zhong W, Xing M (2018). 3D bioprinting for biomedical devices and tissue engineering: a review of recent trends and advances. Bioact Mater.

[17] Ozbolat I, Hospodiuk M (2016). Current advances and future perspectives in extrusion-based bioprinting. Biomaterials.

[18] Gu B, Choi D, Park S, Kim M, Kang C, Kim C. 3-dimensional bioprinting for tissue engineering applications. Biomater Res. 2016;20(1). doi:10.1186/s40824-016-0058-2 10.1186/s40824-016-0058-2 PMC4843207 27114828

[19] Kačarević Ž, Rider P, Alkildani S (2018). An introduction to 3D bioprinting: possibilities, Challenges and Future Aspects. Materials.

[20] Waasdorp R, van den Heuvel O, Versluis F, Hajee B, Ghatkesar M (2018). Accessing individual 75-micron diameter nozzles of a desktop inkjet printer to dispense picoliter droplets on demand. RSC Adv.

[21] Murphy S, Atala A (2014). 3D bioprinting of tissues and organs. Nat Biotechnol.

[22] Zhu W, Ma X, Gou M, Mei D, Zhang K, Chen S (2016). 3D printing of functional biomaterials for tissue engineering. Curr Opin Biotechnol.

[23] Wu G, Hsu S (2015). Review: polymeric-based 3D printing for tissue engineering. J Med Biol Eng.

[24] Koch L, Gruene M, Unger C, Chichkov B (2013). Laser assisted cell printing. Curr Pharm Biotechnol.

[25] Keriquel V, Oliveira H, Rémy M, et al. In situ printing of mesenchymal stromal cells, by laser-assisted bioprinting, for in vivo bone regeneration applications. Sci Rep. 2017;7(1). 10.1038/s41598-017-01914-x. 10.1038/s41598-017-01914-x PMC5431768 28496103

[26] O'Brien C, Holmes B, Faucett S, Zhang L (2015). Three-dimensional printing of nanomaterial scaffolds for complex tissue regeneration. Tissue Eng B Rev.

[27] Williams C, Malik A, Kim T, Manson P, Elisseeff J (2005). Variable cytocompatibility of six cell lines with photoinitiators used for polymerizing hydrogels and cell encapsulation. Biomaterials.

[28] Scranton AB, Bowman CN, Peiffer RW. Photopolymerization. ACS Symp Ser. 1997;673.

[29] Gosavi S, Gosavi S (2010). Local and systemic effects of Unpolymerised monomers. Dent Res J (Isfahan).

[30] Knowlton Stephanie, Yenilmez Bekir, Anand Shivesh, Tasoglu Savas (2017). Photocrosslinking-based bioprinting: Examining crosslinking schemes. Bioprinting.

[31] Hersel U, Dahmen C, Kessler H (2003). RGD modified polymers: biomaterials for stimulated cell adhesion and beyond. Biomaterials.

[32] Tirella A, Liberto T, Ahluwalia A (2012). Riboflavin and collagen: new crosslinking methods to tailor the stiffness of hydrogels. Mater Lett.

[33] Croome R (2007). The variation of the viscosity of gelatin sols with temperature. J Appl Chem.

[34] Yue K, Trujillo-de Santiago G, Alvarez M, Tamayol A, Annabi N, Khademhosseini A (2015). Synthesis, properties, and biomedical applications of gelatin methacryloyl (GelMA) hydrogels. Biomaterials.

[35] Xu X, Jha A, Harrington D, Farach-Carson M, Jia X (2012). Hyaluronic acid-based hydrogels: from a natural polysaccharide to complex networks. Soft Matter.

[36] Kuss M, Kim J, Qi D (2018). Effects of tunable, 3D-bioprinted hydrogels on human brown adipocyte behavior and metabolic function. Acta Biomater.

[37] Duan B, Hockaday L, Kapetanovic E, Kang K, Butcher J (2013). Stiffness and adhesivity control aortic valve interstitial cell behavior within hyaluronic acid based hydrogels. Acta Biomater.

[38] Pereira R, Sousa A, Barrias C, Bártolo P, Granja P (2018). A single-component hydrogel bioink for bioprinting of bioengineered 3D constructs for dermal tissue engineering. Mater Horiz.

[39] Di Biase M, Saunders R, Tirelli N, Derby B (2011). Inkjet printing and cell seeding thermoreversible photocurable gel structures. Soft Matter.

[40] Batchelor R, Kwandou G, Spicer P, Stenzel M (2017). (−)-riboflavin (vitamin B2) and flavin mononucleotide as visible light photo initiators in the thiol–ene polymerisation of PEG-based hydrogels. Polym Chem.

[41] Gopinathan J, Noh I. Recent trends in bioinks for 3D printing. Biomater Res. 2018;22(1). 10.1186/s40824-018-0122-1. 10.1186/s40824-018-0122-1 PMC5889544 29636985

[42] Diamantides N, Wang L, Pruiksma T (2017). Correlating rheological properties and printability of collagen bioinks: the effects of riboflavin photocrosslinking and pH. Biofabrication.

[43] Serna J, Florez S, Talero V, Briceño J, Muñoz-Camargo C, Cruz J (2019). Formulation and characterization of a SIS-based Photocrosslinkable bioink. Polymers (Basel).

[44] Jang J, Kim T, Kim B, Kim S, Kwon S, Cho D (2016). Tailoring mechanical properties of decellularized extracellular matrix bioink by vitamin B2-induced photo-crosslinking. Acta Biomater.

[45] Kim Y, Majid M, Melchiorri A, Mikos A (2018). Applications of decellularized extracellular matrix in bone and cartilage tissue engineering. Bioeng Transl Med.

[46] Crapo P, Gilbert T, Badylak S (2011). An overview of tissue and whole organ decellularization processes. Biomaterials..

[47] McCall A, Kraft S, Edelhauser H (2010). Mechanisms of corneal tissue cross-linking in response to treatment with topical riboflavin and long-wavelength ultraviolet radiation (UVA). Invest Opthalmol Vis Sci.

[48] Partlow B, Applegate M, Omenetto F, Kaplan D (2016). Dityrosine cross-linking in designing biomaterials. ACS Biomater Sci Eng.

[49] Fancy D, Kodadek T (1999). Chemistry for the analysis of protein-protein interactions: rapid and efficient cross-linking triggered by long wavelength light. Proc Natl Acad Sci.

[50] Xu L, Zhong N, Xie Y, Huang H, Jiang G, Liu Y (2014). Synthesis, characterization, in vitro cytotoxicity, and apoptosis-inducing properties of ruthenium(II) complexes. PLoS One.

[51] Elvin C, Vuocolo T, Brownlee A (2010). A highly elastic tissue sealant based on photopolymerised gelatin. Biomaterials..

[52] Al-Abboodi A, Zhang S, Al-Saady M, Ong J, Chan P, Fu J (2019). Printing in situ tissue sealant with visible-light-crosslinked porous hydrogel. Biomed Mater.

[53] Yang Y, Choi Y, Jung D (2013). Production of a novel silk-like protein from sea anemone and fabrication of wet-spun and electrospun marine-derived silk fibers. NPG Asia Mater.

